# Impact of MR-safe headphones on PET attenuation in combined PET/MRI scans

**DOI:** 10.1186/s13550-016-0178-7

**Published:** 2016-03-02

**Authors:** Florian Büther, Alexis Vrachimis, Anne Becker, Lars Stegger

**Affiliations:** Department of Nuclear Medicine, University Hospital Münster, Albert-Schweitzer-Campus 1, 48149 Münster, Germany; Department of Clinical Radiology, University Hospital Münster, Albert-Schweitzer-Campus 1, 48149 Münster, Germany

**Keywords:** PET/MRI, Attenuation correction, MR headphones, Artefacts

## Abstract

**Background:**

MR headphones are attenuation sources affecting PET quantification in hybrid PET/MRI. Despite potentially better patient communication, usage in PET/MRI scans is not approved by the vendor. This study aims to determine the impact of headphones on PET by means of phantom and patient scans. Additionally, the perceived benefit of using headphones was evaluated.

**Findings:**

A cylinder phantom was scanned without and with dedicated MR headphones in a PET/CT scanner. Headphone attenuation was additionally assessed in a clinical setup in 10 patients on a PET/MR scanner using F-18-fluoro-deoxy-glucose. The difference in tracer uptake with and without headset was determined for the various brain regions. Additionally, the patients were asked for differences in noise levels, patient comfort, communication quality, and preference. CT data revealed headphone attenuation values of 350–500 HU. Neglecting headphone attenuation leads to a decrease in PET values between the earcups of about 11 % when compared to the correctly reconstructed data. Regions further away from the headphones were less affected. Patient images demonstrated a decrease of 11 % on average in the cerebellum and temporal lobes, while other regions were less affected. No visual artefacts in the images were noticed. On average, no advantage in terms of noise and patient comfort and only slightly better quality of communication were imparted by the patients.

**Conclusions:**

Using headphones during PET/MR acquisition leads to a negative bias in brain uptake values without introducing obvious image artefacts. Since they lack benefits for the patients, they should be avoided if PET quantification of the brain is needed.

## Findings

### Background

Combined positron emission tomography/magnetic resonance imaging (PET/MRI or PET/MR) systems are becoming more widely available for diagnostic imaging [1]. In contrast to hybrid PET/CT systems, combining PET with computer tomography, there are still shortcomings in present PET/MR scanner designs, especially concerning the attenuation correction of PET data [2–5]. While CT images represent information about attenuation of objects inside the scanner (albeit at lower X-ray energies than the 511 keV photons of PET), MR image contrasts are not related to photon attenuation at all. More complex algorithms are therefore needed to derive attenuation maps from MR data [6–9].

Specific hardware components of the PET/MR system such as coils or the patient bed are usually not visible in MR images. They have to be either of low enough density to be neglected or incorporated as templates into the PET attenuation map [10]. This is a challenging task for hardware components that are not fixed in a specific position [11, 12], e.g. MR-safe headphones.

MR headphones are of value in MRI scanning due to their noise-suppressing and communication-enhancing capabilities. Currently, the influence of headphones on PET attenuation is not accounted for in PET image reconstruction. Therefore, the use of headphones is not approved by the manufacturer for PET image acquisition of the head. However, the headphones may otherwise be of help in whole-body scans, thus justifying their delivery with current PET/MR systems.

In this study, we investigated the effects of additional attenuation caused by headphones on PET brain image quality and quantification, both in phantom and patient scans. Furthermore, we aimed to determine the patients’ subjective assessment of headphones during scanning.

### Methods

**Fig. 1:**
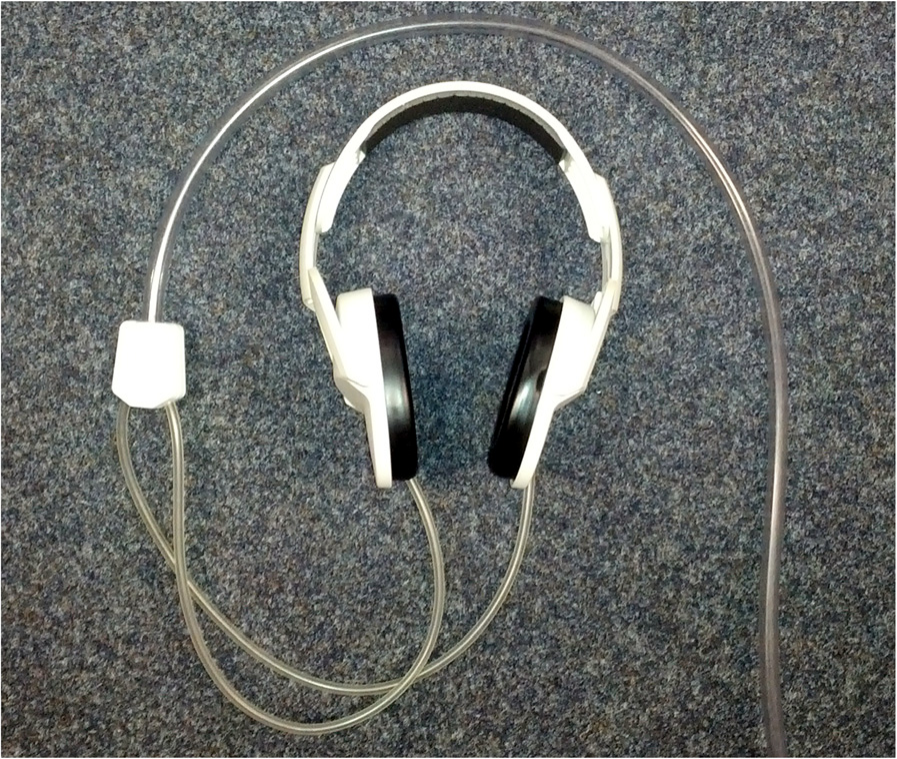
Headphones used in this study consisting of two earcups connected by a flexible headband and air-filled plastic tubes

Standard MR headphones consisting of two earcups connected by a headband and delivered with the mMR PET/MR system (Siemens Healthcare GmbH, Erlangen, Germany) [1] were used throughout this study (Fig. 1). For MR safety, these headphones do not have metallic parts; instead, acoustic information is transmitted by an air pressure system via plastic tubes.

To assess the attenuation properties of the headphones, a standard germanium-68-filled cylinder phantom, used for quality control and normalisation, was scanned twice in the mCT PET/CT scanner (Siemens Healthcare GmbH, Erlangen, Germany) in identical positions: first without headphones then with the headphones placed around the cylinder. CT data of both configurations were acquired at 120 kVp. PET data was acquired for 30 min in one bed position for both headphone configurations. PET image reconstruction (TOF-OSEM 3i21s) was performed with either of the two CT datasets (with and without headphones) used for attenuation correction. Regional PET image value differences between the two data sets were subsequently analysed.

Ten patients (58 ± 16 years) without known disorders of brain glucose metabolism were scanned on the mMR PET/MR system immediately after a clinical PET/CT examination using the radiotracer F-18-fluoro-deoxy-glucose (FDG). A PET-compatible head coil delivered with the PET/MR system was used to acquire MR images for PET attenuation correction with and without the headphones in place (5-min PET scan in one bed position, additional earplugs, approximately 90 min after injection of 4 MBq/kg, OSEM 3i21s with MR-based attenuation and scatter correction). To avoid bias caused by tracer kinetics, the scan without headphones was performed first in five patients and second in another five patients. The difference in start time of both scans was 9.0 ± 0.7 min for patients wearing the headphones during the first scan (range 9.0–11.0 min), and 10.3 ± 1.2 min for patients wearing the headphones during the second scan (range 9.0–12.0 min). During the PET scans, T1- and T2-weighted FLASH (fast low-angle shot) sequences were performed to simulate typical noise levels. During both acquisitions, patients were given instructions through the speaker system/headphones, in order to test communication quality. Afterwards, patients were asked to answer a questionnaire, consisting of the following questions for both scans:

1. Which noise level did you experience during the scan? (1: very low, to 5: very high)
2. How relaxed did you feel during the scan? (1: very relaxed, to 5: very unrelaxed)
3. How well did you hear what the technician said during the scan? (1: very well, to 5: very badly)
4. In future scans, would you choose to use the headphones again? (1: no, definitely not, to 5: yes, definitely, with three denoting indifference)

Tracer uptake in the various brain structures were quantified using the standard brain atlas of the MI-Neurology tool of the syngo.via software (Siemens Healthcare GmbH, Erlangen, Germany), allowing the assessment of the relative mean tracer uptake of the brain regions as referenced in Table 1. The actual region-of-interest definitions are based on registering the PET images on a MR-derived brain atlas [13].

**Table 1:** Decrease in regional tracer uptake between scans without and with headphones

Region	Decrease in uptake (%)
Cerebellum	10.8 ± 1.8
Mesial temporal lobe	8.8 ± 3.2
Temporal lobe	8.8 ± 4.3
Occipital lobe	7.5 ± 2.6
Fissura calcarina	6.8 ± 2.3
Basal ganglia	5.2 ± 2.7
Frontal lobe	4.2 ± 3.0
Gyrus cinguli	3.5 ± 2.7
Parietal lobe	3.3 ± 2.9
Central region	2.5 ± 2.2

Values are given as mean ± standard deviation

### Results

CT images of the cylinder phantom with the headphones in place revealed homogeneous X-ray attenuation of the headphone material of 350–500 Hounsfield units (HU; for comparison, water 0 HU; compact bone 1000–1500 HU; Fig. 1), corresponding to linear absorption values *μ* at 511 keV of around 0.12 cm^−1^, as obtained from the machine-derived PET attenuation images [14]. PET annihilation radiation traversing both earcups (thickness ~2 cm) perpendicularly was therefore expected to decrease in intensity to a value of exp(–0.12 cm^−1^ × 2 cm × 2) = 62 %. This was confirmed by forward projecting the attenuation map using the open source program STIR [15], resulting in a value of approximately 59 % for those lines.

**Fig. 2:**
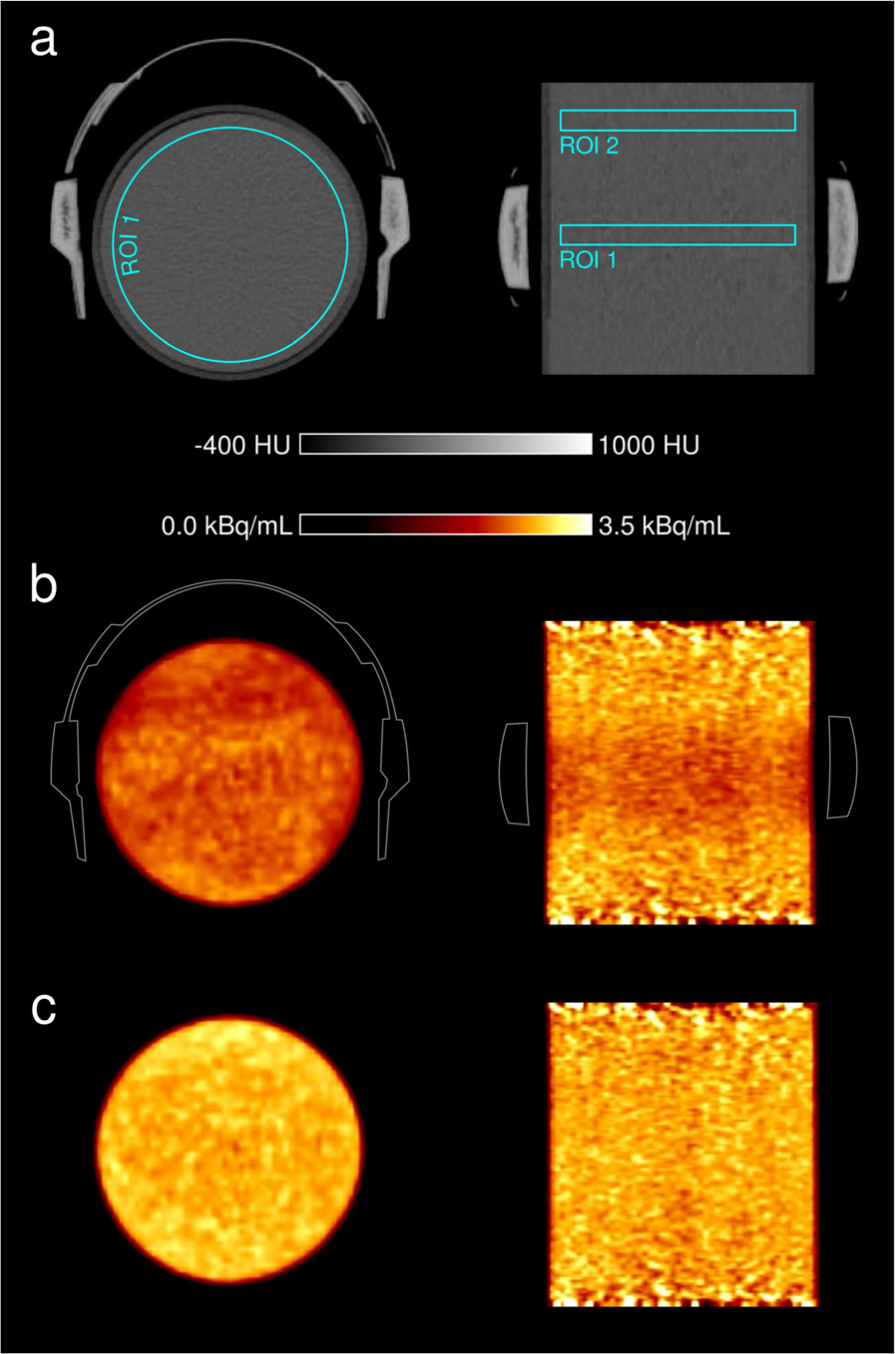
Axial and coronal PET/CT images of the cylinder phantom: CT scan (**a**), PET scan with headset but without headphone attenuation correction (**b**, with contour of headphones), and PET scan with headphone attenuation correction (**c**). Analysed cylindrical regions of interest are depicted as *ROI 1* and *ROI 2*

The reconstructed PET images demonstrated a slightly inhomogeneous decrease in activity values in the headphone region when CT data without headphones were used to correct PET data with the headphones in place as compared to the reference image without headphones in either scan (Fig. 2). On average, a cylindrical region of interest (ROI) between the earcups (cylindrical ROI 1 in Fig. 2) dropped in mean activity by 12 % as compared to images without the headset being present. Taking headphone attenuation into account, the mean activity of ROI 1 was slightly increased by 2 % as compared to the reference image. Regions further away from the headphones were practically not affected (Fig. 2, cylindrical ROI 2, mean activity difference to reference image <1 % for both images).

**Fig. 3:**
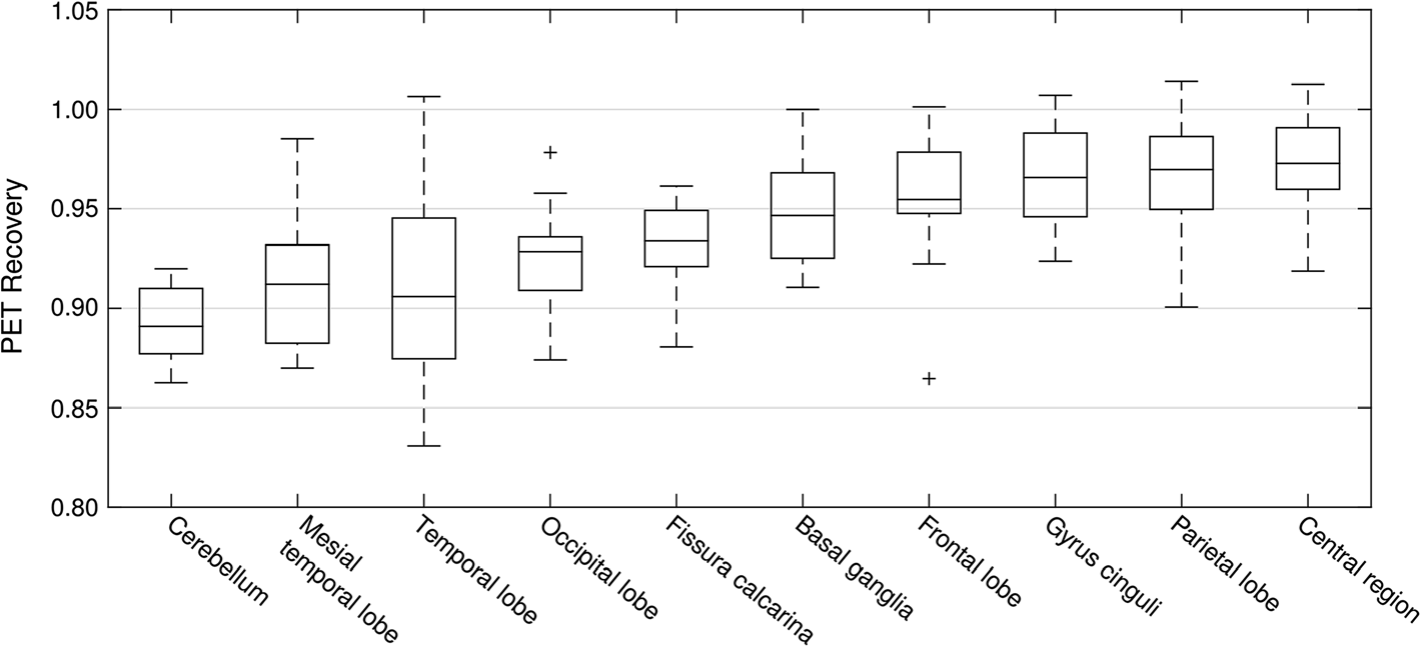
Regional brain uptake analysis in 10 patients, given as PET recovery values (equals mean region uptake in scan with headphones divided by the mean region uptake in scan without headphones). *Plus sign* denotes outliers

**Fig. 4:**
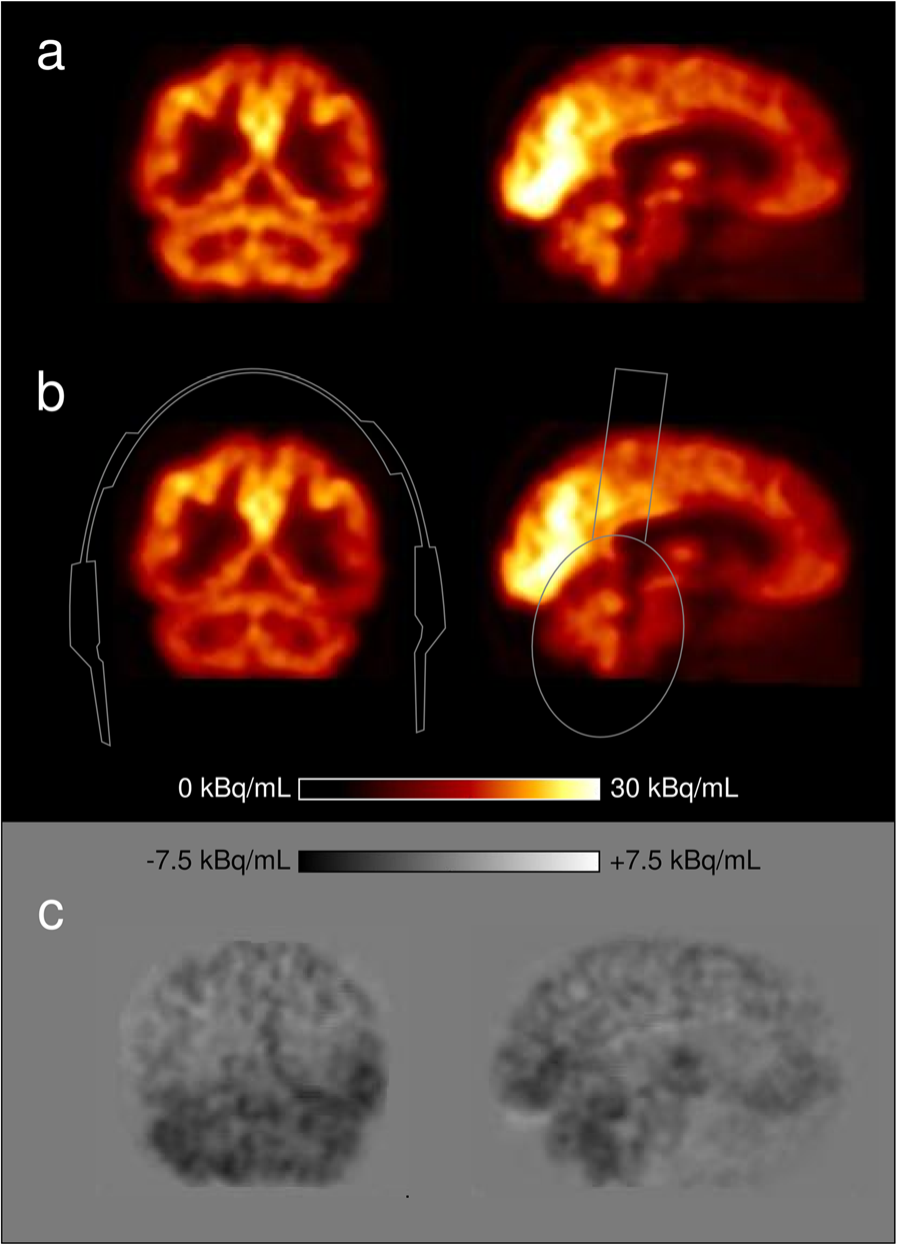
Coronal and sagittal FDG-PET/MR images without (**a**) and with (**b**, approximate position indicated by contour) headphones. Difference image (**c**) reveals a substantial decrease in image values

Regional brain analysis in mean standardised uptake values (SUV_mean_: mean regional activity normalised to injected activity, body weight, and time between injection and scan) of the patient scans with the headphones in place revealed clear underestimation of activities in the cerebellum (decrease 11 % on average) and the temporal lobes (decrease 9 %; Table 1, Fig. 3). All other regions were also underestimated on average, but to a smaller degree (e.g. parietal lobes, 3 %; frontal lobes, 4 %). All differences were found to be significant (*p* < 0.0005 for all regions, Wilcoxon signed-rank test). The decrease of tracer activity was smooth, and no localised visual artefacts in the images were seen (Fig. 4).

Questionnaire answers demonstrated that subjective noise levels experienced by the patients were almost the same in both scans (mean ± standard deviation without headphones 3.3 ± 1.1 vs. with headphones 3.1 ± 1.0, i.e. medium noise levels; *p* = 0.81, Wilcoxon signed-rank test). Similarly, there was no difference in subjective relaxation state (1.7 ± 0.9 vs. 1.6 ± 0.7, i.e. quite relaxed; *p* = 1.0). Quality of communication was judged slightly worse without headphones (2.8 ± 1.5, i.e. satisfying, vs. 2.0 ± 1.0, i.e. good; *p* = 0.22), while the future choice of scanning either with or without headphones was indifferent (2.7 ± 1.2; *p* = 0.44, against total indifference, i.e. a value of 3.0).

### Discussion

The provided headphones were proven to cause considerable attenuation of 511 keV photons of PET, thus justifying the manufacturer’s disapproval of using them for simultaneous PET scanning of the head. Their effects on PET quantification in phantom and patient studies were clearly evidenced in the scans. In the latter, especially the cerebellum and the temporal lobes as regions that are located right between the earcups experienced a rather uniform decrease in uptake values of approximately 10 %, while more remote regions were less affected. Therefore, specifically in PET studies where absolute uptake quantification is necessary (e.g. kinetic modelling of tracer uptake in the brain), the headphones should be avoided. In cases where quantification is not of importance, the headphone-introduced bias may still have diagnostic impact despite the absence of localised visual artefacts.

Surprisingly, the study showed that the subjective benefit for the patients with the headphones in place is small. The noise-cancelling properties of the earplugs and additional rubber foam spacers around the patient’s head may explain this. Furthermore, wearing headphones may add to patient discomfort, especially if they get displaced during the scan. Communication improved slightly, yet not to a degree that made patients choose the headphones if given the choice, as evidenced by the results of the questionnaire.

Nevertheless, some specific applications may require the use of headphones during PET/MR scans (e.g. fMRI scans with auditory stimuli). For these scans, small in-ear headphones could be an interesting alternative as they lack significant attenuation due to their size. MR-safe implementations are already available from third-party vendors, allowing both noise protection and sound conduction. Alternatively, headphone location and orientation may be deduced from MR images using either MR-visible markers or ultra-short TE (UTE) sequences. A template-based approach could then be used to incorporate headphone attenuation into PET image reconstruction.

### Conclusion

Usage of standard MR headphones during simultaneous PET/MR imaging leads to a significant underestimation of tracer uptake in parts of the brain and offers no significant advantages for the patients’ subjective experience. These headphones should therefore be avoided during PET/MR acquisitions. Incorporation of headphone attenuation in the image reconstruction process does not seem to be a high priority for standard PET/MRI.

#### Ethics, consents, and permissions

All procedures performed in this study were in accordance with the ethical standards of the institution and with the principles of the 1964 Declaration of Helsinki and its later amendments. Informed consent was obtained from all participants. The Joint Research Ethics Committee of the Faculty of Medicine, University of Münster, and the locoregional Chamber of Physicians of Westfalen-Lippe approved this prospective study.

#### Consent to publish

Consent to publish was obtained from all participants.

## Abbreviations

MR: magnetic resonance
MRI: magnetic resonance imaging
PET: positron emission tomography
CT: computerised tomography
FDG: F-18-fluoro-deoxy-glucose
SUV: standardised uptake value
fMRI: functional magnetic resonance imaging
TE: echo time
UTE: ultra-short TE
